# Transactions of the Medical Society of Pennsylvania

**Published:** 1852-10

**Authors:** 


*Transactions of the Medical Society of the State of Pennsylvania, May* 1852, vol. ii.—The reports are eminently practical and exhibit much industry and talent.

